# Reply to Fabre et al. Comment on “Tanmoy et al. CRISPR-Cas Diversity in Clinical *Salmonella enterica* Serovar Typhi Isolates from South Asian Countries. *Genes* 2020, *11*, 1365”

**DOI:** 10.3390/genes12081147

**Published:** 2021-07-28

**Authors:** Arif Mohammad Tanmoy, Chinmoy Saha, Mohammad Saiful Islam Sajib, Senjuti Saha, Florence Komurian-Pradel, Alex van Belkum, Rogier Louwen, Samir Kumar Saha, Hubert P. Endtz

**Affiliations:** 1 Department of Medical Microbiology and Infectious Diseases, Erasmus University Medical Center Rotterdam, 3015 CN Rotterdam, The Netherlands; arif.tanmoy@chrfbd.org (A.M.T.); c.saha@erasmusmc.nl (C.S.); h.p.endtz@erasmusmc.nl (H.P.E.); 2 Child Health Research Foundation, 23/2 SEL Huq Skypark, Block-B, Khilji Rd, Dhaka 1207, Bangladesh; saiful.sajib@chrfbd.org (M.S.I.S.); senjutisaha@chrfbd.org (S.S.); samir@chrfbd.org (S.K.S.); 3 Laboratoire des Pathogènes Emergents, Fondation Mérieux, Centre International de Recherche en Infectiologie (CIRI), INSERM U1111, 69365 Lyon, France; florence.pradel@fondation-merieux.org; 4 Data Analytics Unit, bioMérieux, 3, Route de Port Michaud, 38390 La Balme Les Grottes, France; alex.vanbelkum@biomerieux.com; 5 Bangladesh Institute of Child Health, Dhaka Shishu Hospital, Dhaka 1207, Bangladesh

We respectfully thank Fabre et al. for presenting an elaborate discussion on our previously published findings regarding the organization and composition of CRISPR loci in clinical isolates of *Salmonella enterica* serovar Typhi [1]. We presented our data and methods in great detail in the original article to ensure that readers can re-analyze the data or perform similar studies for other pathogens. 

Fabre et al. noted a quality issue with the data presented in our previous article [2]. The authors notified us of this issue soon after our original article on *S.* Typhi CRISPRs was published [1]. We had several email exchanges with Fabre et al., shared the corrected accessions, and informed them about our determination to correct those on public record. We eventually submitted an “Author Correction” to the original paper [1], which is now published and publicly available [3]. The fact that Fabre et al. still raised a point based on the uncorrected dataset surprised us given that they were cognizant of the issue. We, therefore, do not consider the commentary on this topic very constructive.

Fabre et al. also discussed the issue of possible contamination in our genome data [1,2]. The concerned isolates have been described with the complete dataset in our previous article [2]. However, the complete genome dataset was checked for contamination as described [2], using Kmerfinder and SeqSero to confirm the *Salmonella* species and the serovar nature, respectively [2,4,5,6]. Based on that, we discarded three genomes (from the original 539) and proceeded with the analyses of the remaining 536 genomes under discussion [2]. The same dataset was used in our original article on *S.* Typhi CRISPRs [1]. Thus, the scientific rationale behind rechecking the serovar information remains elusive to us. 

We appreciate the efforts made by Fabre et al. to generate a detailed comparative analysis between their ten-year-old data analysis and the new information presented in our recent paper [1]. The field would undoubtedly benefit from an updated definition and associated nomenclature of CRISPR loci for this serovar. It must also be taken into consideration that due to the diversity in current bioinformatic pipelines, differences in the ultimate data interpretation are unavoidable [7]. The differences between the analyses done by Fabre et al. and our findings are interesting and should be further investigated through molecular genetics and functional biochemical studies. However, we do not see a clear scientific rationale to extend this discussion with Fabre et al. at this stage. We are grateful to Fabre et al. for their constructive criticism, and we encourage the readers of this journal to take notice of the topics under discussion and eventually to develop a more consensus-driven approach for CRISPR classification in *Salmonella enterica* serovar Typhi.
